# Restoration of forearm supination by combining pronator teres with allogeneic tendon

**DOI:** 10.1186/s12891-021-04692-w

**Published:** 2021-09-29

**Authors:** Shi-Li Ding, Qianjun Jin, Ji-Hua Xu, Yimeng Liu, Xin Huang, Kejiong Liang, Hai-Fei Shi

**Affiliations:** 1 Department of Orthopedics, The First Affiliated Hospital, College of Medicine, Zhejiang University, #79 Qingchun Road, Hangzhou, Zhejiang Province 310003 People’s Republic of China; 2 Goldsmiths University of London, London, UK

**Keywords:** Supination, Pronator teres, Allogeneic tendon, Radial nerve, Reconstruction

## Abstract

**Background:**

Many solutions have been proposed in treating of forearm supination. Comparing with other supination function reconstructions, pronator teres rerouting is believed to be less effective due to its insufficient supination strength. The aim of this study is to introduce a modified procedure, and compare its result with two previous approaches.

**Patients and methods:**

From 2015 to 2020, 11 patients have restored forearm supination by rerouting of the pronator teres weave sutured with allogeneic tendons. The average follow-up period was 17.5 months (12 to 24). The range of active supination at the final follow-up was recorded.

**Results:**

Almost all patients acquired good supination range. The average active post-operative supination was 72.7° (60° to 80°) at the final follow-up. No complication was observed. All patients retained full range of pronation.

**Conclusions:**

This study provides a modified supination function reconstruction with simple operating, fine results, low risks, and no affecting of pronation function. The use of allogeneic tendon makes up for the muscles with insufficient length, making it valuable to reconsider those rebuilding operations that were once considered unpromising by many.

## Background

Elbow radial nerve injuries do not usually cause loss of supination, because bicep is a strong supinator. But biceps brachii can only produce the greatest force when the angle of elbow flexion is between 90° and 120°. Muscle strength decreases gradually as the elbow moves farther away from this angle [1]. When the elbow is at a 90° angle, supination strength is at its greatest, whereas supination strength decreases as the elbow approaches 0° due to the contribution of biceps brachii to the supination movement [2]. Meanwhile, the biceps generate most of their torque while in pronation, and may not produce supination beyond the neutral position of the forearm if not assisted by a functioning supinator [3]. For the reasons above, some patients who suffer from elbow radial nerve injuries without receiving special supination function reconstruction, complain about their insufficient supination function after treatment.

Due to the short tendinous portion of pronator teres, the traditional pronator teres rerouting through interosseous membrane to restore supination failed to provide enough torsion. Its application is limited. In our practice, an allogeneic tendon is woven into the distal end of pronator teres to increase its length, and the insertion is relocated thereafter. Then the supination function was effectively restored.

## Patients and methods

All participants fit in the following criteria: received radial nerve anastomosis for 6 months to 2 years with no satisfactory supination; forearm supination played a major role in daily life or work; forearm pronation strength reached grade 5 on MRC muscle scale; complete passive forearm supination (mean 83°, 75° to 90°) can be achieved (Table 1); no special supination function reconstruction was received. Patients were assessed monthly for the first 3 months after surgery, thereafter twice annually. The range of active supination at the final follow-up was recorded.

**Table 1:** Summary of patients

Cases	Age (yrs)	Sex	Side	Passive pre-operative supination (°)^a^	Active post-operative supination (°)	Follow-up (mths)
1	33	M	R	80	80	20
2	52	M	R	85	75	12
3	50	F	L	75	60	24
4	45	M	L	90	80	18
5	23	M	R	90	75	24
6	36	F	L	80	70	12
7	37	M	R	80	65	24
8	33	M	L	90	80	12
9	44	M	R	75	70	17
10	51	M	R	80	75	13
11	49	F	R	85	70	16

^a^all the patients have no active pre-operative supination (0°)

### Statistics

The increased active supination angle (the angular difference between pre- and post-operative active supination angle) were calculated. Then one-way ANOVA test was used to analyze the difference between our result, Aderson’s [4], and Amrani’s [5] work. Statistical calculation was performed with SPSS 24.0 software (Chicago, Illinois). The differences with a *P* value less than 0.05 were considered as statistically significant.

### Surgical technique

**Fig. 1:**
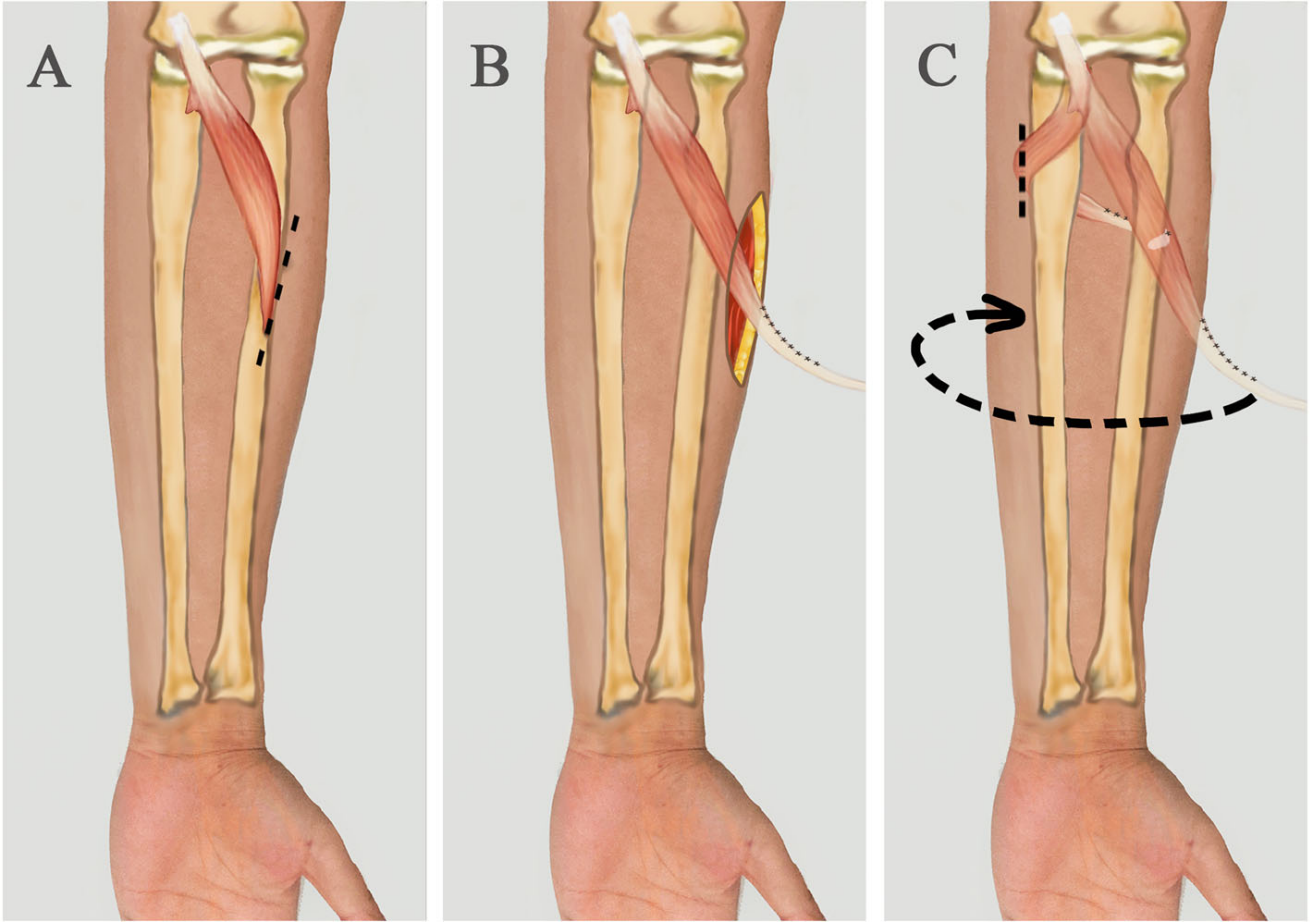
A short longitudinal incision is made to expose the distal insertion of pronator teres (**a**). The pronator teres is released and lengthened with an allogeneic tendon (**b**). The tendon is pulled to the palmar ulna under flexor digitorum profundus. Bypassing the ulna, the tendon is pulled back under the extensor digitorum muscle to the radialis side. Around the radius, on the radial palmar side of the radius, the insertion is rebuilt at the original insertion of pronator teres using 1.8 mm suture anchor (**c**)

A short longitudinal incision is made at the radial palmar midforearm to expose the distal insertion of pronator teres (Fig. 1a. Then the distal pronator teres is released from the radius. Tendinous portion of distal pronator teres is lengthened by stitching with an allogeneic tendon of the same thickness using the modified Kessler tendon suture technique and 3–0 Ethibond suture (Ethicon Inc., Somerville, NJ) (Fig. 1b). Make a longitudinal auxiliary incision on the ulnar side of the forearm, pass the tendon under the flexor digitorum profundus, and lead out from the auxiliary incision. Bypassing the ulna, the tendon is pulled back under the extensor digitorum muscle to the radialis side. Around the radius, on the radial palmar side of the radius, the insertion is rebuilt at the original insertion of pronator teres using 1.8 mm suture anchor (DePuy Mitek, Raynham, MA) (Fig. 1c).

Before finishing the rebuilding, it should be ensured that the tension of pronator teres can hold the forearm to about 45° supination with the elbow in 90° flexion. Among some patients, pronator quadratus and/or interosseous membrane should be released to achieve adequate supination. The sutures are removed 12 to 14 days after surgery. Two demountable over-elbow plasters are applied to keep the elbow at 90° and the forearm in maximum supination for 3 weeks. The patients are encouraged to start initiative non-confrontational supination after the plasters are removed. Weight-bearing exercises can be started 6 weeks after operation.

## Result

**Fig. 2:**
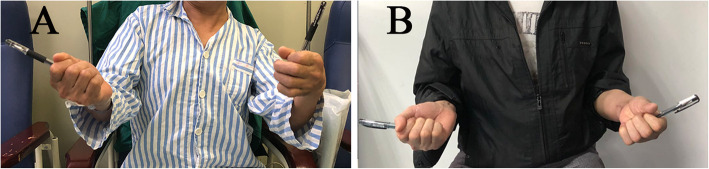
A 52 years old man with a pronation deformity after elbow radial nerve injury. Pre-operatively, the supination angle was 10° (**a**). Six months after operation, with 75° supination (**b**)

From 2015 to 2020, 11 patients with elbow radial nerve injuries underwent this surgery to improve forearm supination function. 8 males and 3 females at an average age of 41.2 (23 to 52) years old participated. No complications were observed. The follow-up period is 12 to 24 months (mean 17.5 months). All patients retained full range of pronation. Almost all patients acquired good supination range (Fig. 2). The average active post-operative supination was 72.7° (60° to 80°) at the final follow-up. Most of our patients were factory workers, they were able to perform daily activities involving forearm supination such as hair combing, using of chopsticks and spoons, twisting handles, reading on books and phones and tightening screws.

The average supination improvement was 72.7 ± 6.47° (60° to 80°) at the final follow-up. The supination improvement in our study was significantly larger than Aderson’s study (37.27 ± 18.21°, *n* = 11, *p* < 0.001). There is no difference in supination improvement between our method and Amrani’s study (73.57 ± 6.33°, *n* = 14, *p* = 0.855).

## Discussion

Aderson et al. [4] transfered the tendon of flexor carpi ulnaris to the split tendon of brachioradialis with its bony insertion into the radial styloid. The average supination improvement was 37.27 ± 18.21°. Amrani et al. [5] corrected the pronation deformity in 14 children by rerouting the distal part of pronator teres dorsally to volarly through a window in the interosseous membrane and suturing to the proximal tendon. The average supination improvement was 73.57 ± 6.33°. Through statistical comparison, we found that average supination improvement of our results were significantly better than the former, and there was no significant difference from the latter.

Sakellarides et al. [6] in 1981 proposed firstly that by modifying the original pronator teres radius insertion to the opposite side to act as supination force, it has resulted in an average of 46° supination. However, the significance of such operation has been doubted over time by scholars. Strecker et al. [7] verified that pronator teres rerouting surgery result in a better supination function than simple tendon lysis. Nevertheless, the research of Veeger et al. claimed that the effect of pronator teres rerouting restoring supination is equivalent to a tendon lysis [8]. Gschwind and Tonkin carried out their modified approach of a Z-shaped prolongation of pronator teres tendon followed by repairing it bypassing the posterior of radius, pronation strength is thereafter released, good postoperative result is obtained [9]. Although rebuilding of tendon insertion is avoided, due to relatively shorter tendon length, the improper handling of tensile strength can still occasionally affect pronation [10, 11]. The resistance to tensile load of scar-healing prolonged tendon is also comparatively weakened.

Although different operation results have been reported, a number of surgeons still adopt the pronator teres rerouting approach to restore supination, especially when wrist and finger extensions are simultaneously in need of rebuilding while flexor carpi ulnaris muscle or other muscles are selected for use. In most studies above, pronator teres run through interosseous membrane instead of subcutis to avoid adhesion. However, the interosseous membrane plays a key role in a series of ligaments which maintains the stabilization of forearm. Injuring interosseous membrane would affect longitudinal and transverse stabilization of forearm [12]. Incomplete interosseous membrane incision would result in entrapment of pronator teres. Improved supination from simply rerouting pronator teres through interosseous membrane is limited on account of windlass effect, making it ineffective in converting muscle force into supination force [13]. The operating area in such surgery involves more significant deep anatomical structure such as anterior interosseous nerve/artery, posterior interosseous nerve/artery. These structures would get more unrecognizable among scarring soft tissue after primary surgery, increasing operational time and difficulty. Now, these problems can be avoided with our approach. Our method avoids damage to the structures between the ulna and radius, reduces the risk of surgery, and simplifies the procedure.

Pronator teres starts from medial epicondyle of humerus and the medial side coronoid process of ulna, crossing the forearm diagonally and inserting halfway down the lateral surface of the radius. Supinator takes its origin from lateral epicondyle of humerus and lateral side of ulna, ending at the upper volar palmar radius. The origin and termination of these two muscles are at close distance and respectively put radius in spinning motion around ulna in opposite directions. Therefore, rerouting pronator teres as supinator is essentially duplicating the mechanism of supinator.

Van Heest et al. [13] meticulously underwent cadaveric studies about restoring of supination through pronator teres rerouting, comparing pronator teres insertions at 6 different positions: volar insertion, interosseous ligament insertion, dorsal insertion, native insertion after rerouting around the radius, volar insertion after rerouting around the radius, and 6 new positions 1 cm shifted toward the near end of radius from their original positions. By studying the 12 insertions, the optimum supination is acquired when pronator teres is rerouted through an interosseous window and reinserted into its original insertion place or onto the volar surface of radius. The average active supination angle is at 47°, with no evident disparity of that with 1 cm shifts toward proximal radius. This insertion is adopted in our method due to its relatively good supination.

## Conclusions

This work provides a modified supination function reconstruction. The supination improvement in our study was significantly larger than Aderson’s study (37.27 ± 18.21°, *n* = 11, *p* < 0.001). There is no difference in supination improvement between our method and Amrani’s study (73.57 ± 6.33°, *n* = 14, *p* = 0.855). The use of allogeneic tendon makes up for the muscles with insufficient length, making it valuable to reconsider those rebuilding operations that were once considered unpromising by many.

## Data Availability

The datasets supporting the conclusions of this article are included within the article. The raw data can be requested from the corresponding author.

## Abbreviations

MRC: Medical research council
SPSS: Statistical product and service solutions
